# *Neospongodes atlantica*, a potential case of an early biological introduction in the Southwestern Atlantic

**DOI:** 10.7717/peerj.14347

**Published:** 2022-12-15

**Authors:** Ralf T.S. Cordeiro, Ágatha Nascimento Carpinelli, Ronaldo B. Francini-Filho, Barbara de Moura Neves, Carlos D. Pérez, Umberto de Oliveira, Paulo Sumida, Henrique Maranhão, Leonardo H.U. Monteiro, Pedro Carneiro, Marcelo V. Kitahara

**Affiliations:** 1 Departamento de Biologia, Universidade Federal Rural de Pernambuco, Recife, Pernambuco, Brazil; 2 Department of Zoology (Invertebrate Zoology), National Museum of Natural History, Smithsonian Institution, Washington, D.C., United States of America; 3 Programa de Pós-graduação em Biodiversidade e Ecologia Marinha e Costeira, Universidade Federal de São Paulo, Santos, São Paulo, Brazil; 4 Centro de Biologia Marinha, Universidade de São Paulo, São Sebastião, São Paulo, Brazil; 5 Department of Fisheries and Oceans, St. John’s, Newfoundland, Canada; 6 Centro Acadêmico de Vitória, Universidade Federal de Pernambuco, Vitória de Santo Antão, Pernambuco, Brazil; 7 Programa de Pós-Graduação em Ecologia, Teoria, Aplicações e Valores, Universidade Federal da Bahia, Salvador, Bahia, Brazil; 8 Departamento de Oceanografia Biológica, Instituto Oceanográfico, Universidade de São Paulo, São Paulo, Brazil; 9 Programa de Pós-Graduação em Oceanografia, Departamento de Oceanografia, Universidade Federal de Pernambuco, Recife, Pernambuco, Brazil; 10 IVIG, COPPE, Universidade Federal do Rio de Janeiro, Rio de Janeiro, Brazil; 11 Grupo Sandmine & Inframar, Fortaleza, Ceará, Brazil; 12 Universidade Federal do Delta do Paranaíba, Parnaíba, Piauí, Brazil

**Keywords:** Octocorallia, Invasion, Continental shelf, Soft-bottom, Habitat Modelling, Brazil

## Abstract

Soft corals (Anthozoa: Octocorallia) are discreet components in the Southwestern Atlantic reef communities. In Brazil, the native octocoral shallow-reef fauna is mostly represented by gorgonians. Consequently, except for the nephtheid *Neospongodes atlantica*, most of the known soft corals from this region are considered non-indigenous. Hitherto, the monotypic genus *Neospongodes*, which was proposed in the early 1900s, has been considered to be endemic to the Northeastern Brazilian coast. Herein, based on *in situ* records, we show that *N. atlantica* is a substrate generalist that has been probably expanding its distribution by dominating extensive shallow and mesophotic sandy and reef bottoms, generally outcompeting other reef benthic organisms, including Brazilian endemic species. Based on previously unidentified museum specimens, new records, and a broad literature review, we provide the most comprehensive modelling of the potential distribution of this species in the Southwestern Atlantic. Based on molecular inference supported by in-depth morphological analysis, the probable non-indigenous and, therefore, ancient introduction of *N. atlantica* in Brazilian waters is discussed. Finally, these results support that *Neospongodes* and the Indo-Pacific *Stereonephthya* are synonyms, which led us to propose the latter as taxonomically invalid.

## Introduction

The Brazilian Exclusive Economic Zone harbors the largest and most diverse coral reef communities in the South Atlantic (Leão, Kikuchi & Testa, 2003; Francini-Filho et al., 2013), including a high percentage of endemic corals (Souza et al., 2017). Brazilian shallow-water reefs have been the focus of most marine assessments to date, including biodiversity surveys and mapping (*e.g.*, Leão et al., 2016). However, in the last decade, ecosystems deeper than 30 m (mesophotic ecosystems) started to be assessed, with most studies concentrated in oceanic islands and the north, northeastern and central Brazilian continental shelfs (Cordeiro et al., 2015; Magalhães et al., 2015; Moura et al., 2016; Pinheiro, Bernardi & Simon, 2017; Francini-Filho et al., 2019; Soares, Tavares & Carneiro, 2018).

Recently, remotely operated vehicle (ROV) surveys have revealed dense monospecific aggregations of the soft coral *Neospongodes atlantica* Kükenthal, 1903 on soft-bottoms off several localities of the northeastern Brazilian coast, a still undescribed kind of benthic community (Moura et al., 2013). *Neospongodes atlantica* is the only representative of the genus and, to date, considered endemic to Brazil (Castro, Medeiros & Loiola, 2010). In terms of distributional records, *N. atlantica* has been reported only to its type locality and the type locality of *N. bahiensis* (see Kükenthal, 1903 - the later synonymized with the former by Verseveldt (1983)), both representing shallow-reefs from the Bahia State (∼12–16°S). One additional single record was that from Castro, Medeiros & Loiola (2010) at the Rio Grande do Norte State (∼6°S). Overall, octocorals are considered ecosystem engineers that, in some localities, support dense assemblages in shallow and deep-waters, adding tri-dimensionality, modulating water flow and, consequently, increasing the diversity in reef environments (Roberts, Wheeler & Freiwald, 2006; Buhl-Mortensen et al., 2010; Nelson & Bramanti, 2020). Octocorals are reported from all oceans and are ubiquitous in coral communities around the globe, being regarded as one of the main components and great competitors for space in benthic assemblages of the Indian and Central Western Pacific ocean basins (Fleury et al., 2008; Janes & Mary, 2012; Pérez et al., 2016), as well as their rise in the Caribbean during the last decades (Lasker et al., 2020).

In the Southwestern Atlantic, more specifically in the Brazilian coast, octocorals are considered discreet components of the reef communities (see Pérez et al., 2016; Aued et al., 2018), with reduced populations (Cassola et al., 2016) usually concentrated in narrow bathymetric and geographic ranges (*e.g.*, Castro, Medeiros & Loiola, 2010; Francini-Filho et al., 2018). In general, the Caribbean and Brazilian octocoral communities are predominantly composed of gorgonian corals (*e.g.*, families Gorgoniidae and Plexauridae) (Bayer, 1961; Pérez et al., 2016; Sánchez, 2016). On the other hand, the Indo-Pacific octocoral communities are dominated by soft corals, mainly those from the families Alcyoniidae, Xeniidae and Nephtheidae (Fabricius, 1997; Fabricius & Alderslade, 2001). Such biogeographical pattern raises uncertainties on the native condition of nephtheids, such as *Neospongodes*, in Brazilian waters.

Although other *Neospongodes* species have been described, it is currently accepted as a monotypic genus (Cordeiro et al., 2021). For example, *Neospongodes agassizi* (Deichmann, 1936) and *Neospongodes caribaea* (Deichmann, 1936) were transferred to the genus *Chironephthya* (Cairns & Bayer, 2009; Imahara et al., 2017), whereas *Neospongodes portoricensis* (Hargitt & Rogers, 1901) is accepted as *Stereonephthya* (Verseveldt, 1983). Because of such taxonomical challenges, it is also important to consider the relationship between the *Neospongodes* and *Stereonephthya*, the latter common in the Indo-Pacific (Fabricius & Alderslade, 2001; Chanmethakul, Chansang & Watanasit, 2010).

Morphologically, *Neospongodes* is almost indistinguishable from *Stereonephthya* in most characters, such as colony and sclerite shape, size, and distribution (Verseveldt, 1983; Ofwegen & Groenenberg, 2007). Also, recent molecular-based phylogenies have added to the previous evidence that there are species complexes that demand more in-depth studies (Ofwegen, 2005; Ofwegen, 2007). Assuming that both *Neospongodes* and *Stereonephthya* correspond to a single genus, its occurrence in the Southwestern Atlantic could represent an ancient introduction. Such hypothesis is tested herein in the light of classical morphology and molecular data. In such a context, since the Brazilian unique marine biodiversity is under crescent threats, such as industrial pollution, fisheries, ocean drilling, and bioinvasion (Werner, Pinto & Dutra, 2000; Miranda & Marques, 2016; Creed et al., 2017; Francini-Filho et al., 2018; Capel et al., 2020; Carpinelli et al., 2020; Tanasovici, Kitahara & Dias, 2020), conservation actions are needed to address such challenges (Castro & Pires, 2001; Picciani et al., 2016). Apart from shedding light on *N. atlantica* taxonomy and systematics, we also modeled its potential habitat (PH) distribution (including the newly found octocoral forest (OF)) through Species Distribution Models (SDMs) (Pearson et al., 2007). Taken together, our results provide the very first tools for decision-makers in terms of the potential invasiveness of this species on the Brazilian coast and discuss on possible ecological consequences of *N. atlantica* silent spread into Brazilian shallow-reef areas.

## Material and Methods

### *In situ* records

Newly discovered and previously reported Brazilian OFs were accessed through non-targeted SCUBA and technical diving, and ROV surveys between 2007 and 2017 in depths of up to 80 m deep at three localities in distinct latitudes. *In situ* records resulted from occasional findings extracted from not-scaled and not-standardized surveys. For this reason, some information regarding dive/mission are missing, including ROV bottom time. The first survey, at the Equatorial outer continental shelf (3°26′S/38°08′W, off Ceará State), occurred in March 2010 at 61 m deep using a Seabotix ROV, revealing a relatively flat rocky substrate covered by a thin veneer of medium grained sands with about 12% of CaCO_3_ (Moura et al., 2013; https://figshare.com/articles/media/Supplemental_Video_1_mp4/21534636). Another survey, carried out at the Cabeço Brasil through technical dives on November 2015 (08°13′S/34°36′W, off Pernambuco State), aimed at a mesophotic coral-sponge community between 50 and 75 m at a continental shelf-break paleo-channel. This paleo-channel displayed a central sandstone reef surrounded by soft-bottoms and rhodoliths (https://figshare.com/articles/media/Supplemental_Video_2_mp4/21539517). Finally, the last sets of *in situ* information gathered using technical and ROV dives were performed at the Abrolhos Bank (17°53′S/18°20′S and 38°40′W/39°10′W, Bahia State) around shallow reef pinnacles (*e.g.*, Timbebas reef) between 18 and 81 m (https://figshare.com/articles/media/Supplemental_Video_3/21539613). Field studies were performed under ICMBio licenses SISBIO 15691-1 and SISBIO-11709-1.

### Museum specimens and morphological analysis

Fifty-nine specimens of *N. atlantica* from the Petrônio Alves Coelho Oceanographic Museum (MOUFPE-CNI) and National Museum (MNRJ) collections (Table S1) were examined to investigate the distribution of OFs in Brazilian waters. Fragments of specimens were dissolved in sodium hypochlorite to allow sclerite examination with a light-microscope and also a scanning electron microscope (*Jeol 6460-LV*). Identifications followed Bayer (1961) and Bayer, Grasshoff & Verseveldt (1983). A specimen sampled through technical diving off Recife (Pernambuco State, Brazil) was used for DNA extraction.

### DNA extraction, amplification and sequencing

Total genomic DNA was extracted using the DNeasy blood and tissue kit (Qiagen, Inc. Valencia, CA, USA) following the manufacturer’s protocol. DNA extraction yield and quality were verified using a spectrophotometer (Nanodrop, Thermo Fisher Scientific, Waltham, MA, USA) and a 1% agarose gel electrophoresis, respectively. Polymerase chain reactions (PCR) cycling of mitochondrial genes followed McFadden et al. (2006) and Cairns & Wirshing (2015), and targeted the genes: *mtMutS* (*msh1*); and *COI + IGR1* (Table 1). The nuclear long ribosomal gene *28S rDNA* was partially amplified according to Halàsz et al. (2015) (Table 1). For each gene, PCR reaction totaled 15 µl containing: 1.5 µl of the 10X PCR buffer; 0.45 µl of MgCl2 (50 mM); 0.3 µl of dNTP (10 mM); 0.3 µl of each primer (10 µM); 11.09 µl of ddH2O; 0.06 µl of Platinum *taq* DNA Polymerase (0.6 U); and 1.0 µl of the DNA sample. Amplicons were purified according to ExoSAP-IT protocol and sequenced using the AB 3500 Genetic Analyzer in both directions. This study is registered under SISGEN record A7E8638.

### Phylogenetic analysis

Sequences were edited and concatenated using Geneious v.2020.2.2 and Fasta Alignment Joiner (Villesen, 2007), and then aligned with nepththeid sequences deposited in GenBank (Table S1) using MUSCLE available at the EMBL-EBI platform (Edgar, 2004; Madeira et al., 2019), and manually refined using Jalview v.2.11.1.0 (Waterhouse et al., 2009). Statistical analyzes were performed using the software MEGA-X (Kumar et al., 2018). *Acrophytum claviger* (GenBank accession: JX203823.1) was used as an outgroup. The JModelTest2 (Darriba et al., 2012) was used to define the best-fit model of evolution of the resulting nucleotide alignment. Bayesian Inference (BI) of the *mtMutS* alignment was performed using MrBayes v.3.2.7 (Ronquist & Huelsenbeck, 2003) at the CIPRES platform (Miller, Pfeiffer & Schwartz, 2010) with four parallel runs with 10 million generations each. The first quarter of the sampled topologies were discarded as burnin. For the concatenated alignment, BI was performed in four parallel runs with 4 million generations each and had the same percentage of the sampled trees discarded as burnin. Maximum-likelihood analysis (ML) was performed using the RAxML v.8.2.12 with 1,000 bootstrap replications (Stamatakis, 2014).

**Table 1:** List of primers utilized to amplify and partially sequence the mitochondrial (*COI, MSH1*) and nuclear (*28S*) gene regions.

**Primers**	**Sequence 5′–3′**	**References**	**Gene**
MSH1-F	AGGAGAATTATTCTAAGTATGG	Herrera, Baco & Sánchez (2010)	*mtMuts*
MSH1-3458R	TSGAGCAAAAGCCACTCC	Sánchez, Lasker & Taylor (2003)	*mtMuts*
CO1-LA-8398F	AATGGCGGGGACAGCTTCGAGTATGTTAATACGG	Brugler & France (2008)	*CO1*
CO1-OCT-R	ATCATAGCATAGACCATACC	France & Hoover (2002)	*CO1*
28S-Far	CACGAGACCGATAGCGAACAAGTA	McFadden & van Ofwegen (2013)	*28S*
28S-Rab	TCGCTACGAGTCTCCACCAGTGTTT	McFadden & van Ofwegen (2013)	*28S*

### Occurrence, environmental data, and modelling

Besides the examined specimens and video surveys, previously published information on occurrence records (latitude and longitude) (Epifanio et al., 1999; Dutra et al., 2006a; Ferreira et al., 2009; Lima et al., 2013) and also from open data sources (*e.g.*, http://splink.cria.org.br and http://gbif.org) were compiled.

Through simple rarefaction, occurrence data were reduced to one per pixel (∼1 km) of the study area. Pixels that showed environmental data values far from the centroid of the overall values (based on the weighted value of each PC result) were excluded from further analyzes using the rarefaction filter for environmental heterogeneity available in SDMtoolbox v.2.2 (Brown et al., 2017). The remaining records were managed in two groups: (i) new records and *in situ* data (model building records); and (ii) existing records (model evaluation records) (ESM 2).

Environmental (predictor) variables were sourced from the bio-oracle database (http://www.bio-oracle.org; Tyberghein et al., 2012; Assis et al., 2018) (Table 2). Bathymetric information was harvested from naturalearthdata.com. These variables were cropped to the study area using the “CropRaster” function of the ENMGadgets R package (Barve & Barve, 2013). Since *N. atlantica* has few documented occurrence records, we assumed a small number of variables as sufficient (Ficetola et al., 2014). Therefore, a Principal Component Analysis (PCA) was performed to check which variables of the environmental dataset had a higher contribution value (*e.g.*, *r* > 0.7).

**Table 2:** Variables used to generate models and respective auto-correlations.

Variables	Correlations
Chlorophyll.max	Primary productivity.max
Dissolved oxygen.min	No correlations
Nitrate.range	Sillicate.range
Primary productivity.max	Chlorophyll.max
Salinity.max	Temperature.max, Temperature.range
Sillicate.range	Nitrate.range
Temperature.max	Salinity.max, Temperature.range
Temperature.range	Sillicate.range, Temperature.max

**Figure 1:**
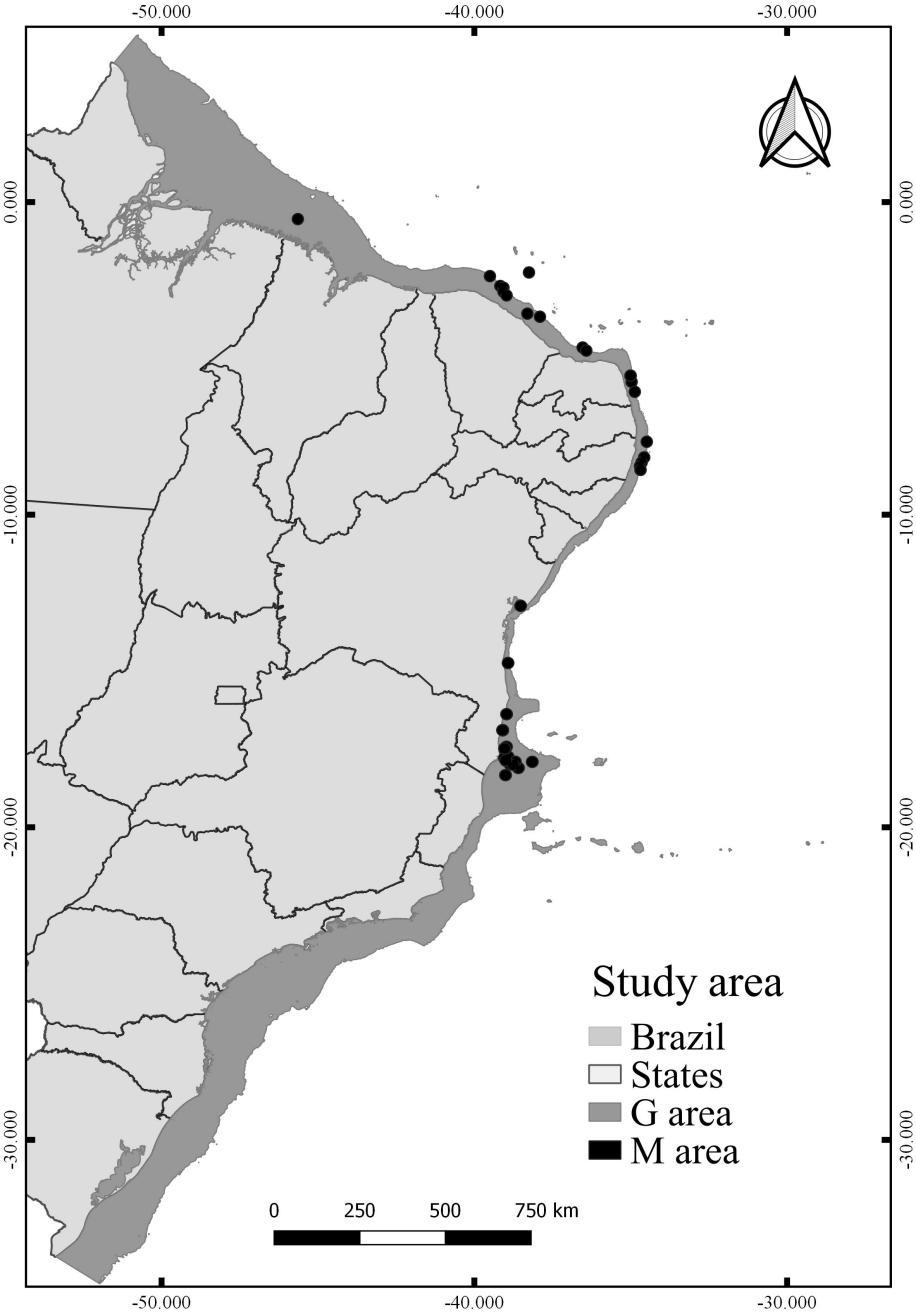
Study area, including the model calibration area (M) and the model projection area (G).

The calibration area for the models (M) corresponds to the entire area to which the species had access at some point (Barve et al., 2011). The choosing of M has been well discussed in the literature (Soberón & Peterson, 2005; Owens et al., 2013), especially to reduce the hypothesis bias regarding the access of species (Anderson & Raza, 2010). Since the known distribution of *N. atlantica* is limited, we extended the area around the occurrence records by 20 km^2^ in diameter, thus creating a perimeter (M) representing only the known accessible area for the species (Fig. 1). This method reduces uncertainties about the distribution of the species and the risk of extrapolating the model to new areas beyond the known distribution (Nuñez Penichet et al., 2021; Fulgêncio-Lima et al., 2021; Machado-Stredel, Cobos & Peterson, 2021). Overall, the model projection area (G) extends along the Brazilian Continental shelf (Fig. 1), from the coastline to the shelf-break (0–200 m).

The Maxent software (Phillips, 2005) was used through the dismo package (Hijmans et al., 2013) to model potential habitats (PH) for *N. atlantica* in the study area, with the Kuenm package (Kükenthal, 1903) applied to find the best parameterization configuration. Candidate models were calibrated using all “feature classes” (FC) combinations available at the Maxent; 29 parameters “Regularization Multiplayer” (RM) (0.1–1 with 0.1 interval and 1–10 with 0,5 intervals), maximum “background” number (100,00), 100 repetitions, and 64 sets of variables.

For each Maxent configuration parameter, two candidate models were created using the “kuenm_val” function, of which one with the complete set of modeling records and the other with 75% of the modeling records. To evaluate candidate models, three criteria were used *via* “kuenm_ceval” function: (i) models built with 75% of the modeling records were evaluated for statistical significance using the “partial ROC tests” (Peterson, Papeş & Soberón, 2008), and then for performance through the omission rate (using 25% of the modeling records); (ii) models constructed with the complete set of modeling records were evaluated using the corrected small sample Akaike Information Criterion (AICc) (Warren, Glor & Turelli, 2010); and (iii) models that passed in both criteria were projected in G. The best model of those projected in G was chosen according to “partial ROC tests” and omission rates (E = 5%), respectively (Kükenthal, 1903).

The final models were created in G through three types of extrapolations: (1) strict extrapolation, in which the models show a response even if the environmental conditions of G are outside the environmental range of the calibration area (M); (2) without extrapolation, in which areas of G with more extreme environmental conditions than those in the calibration area (M) receive zero response; and (3) extrapolation by fixation, which was the one used to calculate the Potential Suitable Habitat (PH). The final models were built using extrapolation by clamping, where areas of G that are environmentally different from M are fixed on the periphery of the environmental region of the calibration area (M) (Kükenthal, 1903), making the model assign a low environmental suitability value to these areas.

To assess the risk of extrapolating the environmental space from M to G, the multivariate environmental distances between the transfer area (G) and the nearest portion of the calibration region (M) were calculated applying the mobility-oriented parity metric (MOP) (Owens et al., 2013) using the “kuenm_mmop” function. The map indicating the places with a high risk of extrapolation (risk of uncertainty) was constructed using the “kuenm_mop” function. Finally, the binary adequation map was built using maximum threshold values, which are more adequate to species with reduced occurrence records (Liu, White & Newell, 2013). All shapefiles were obtained from open sources (IBGE, http://www.naturalearthdata.com), and the maps were built using the Information System and Geoprocessing software (Quantum GIS (Development Team, 2014)). All data used in the modelling analyzes are available as (Data S1–Data S3).

## Results

### In situ records

**Figure 2:**
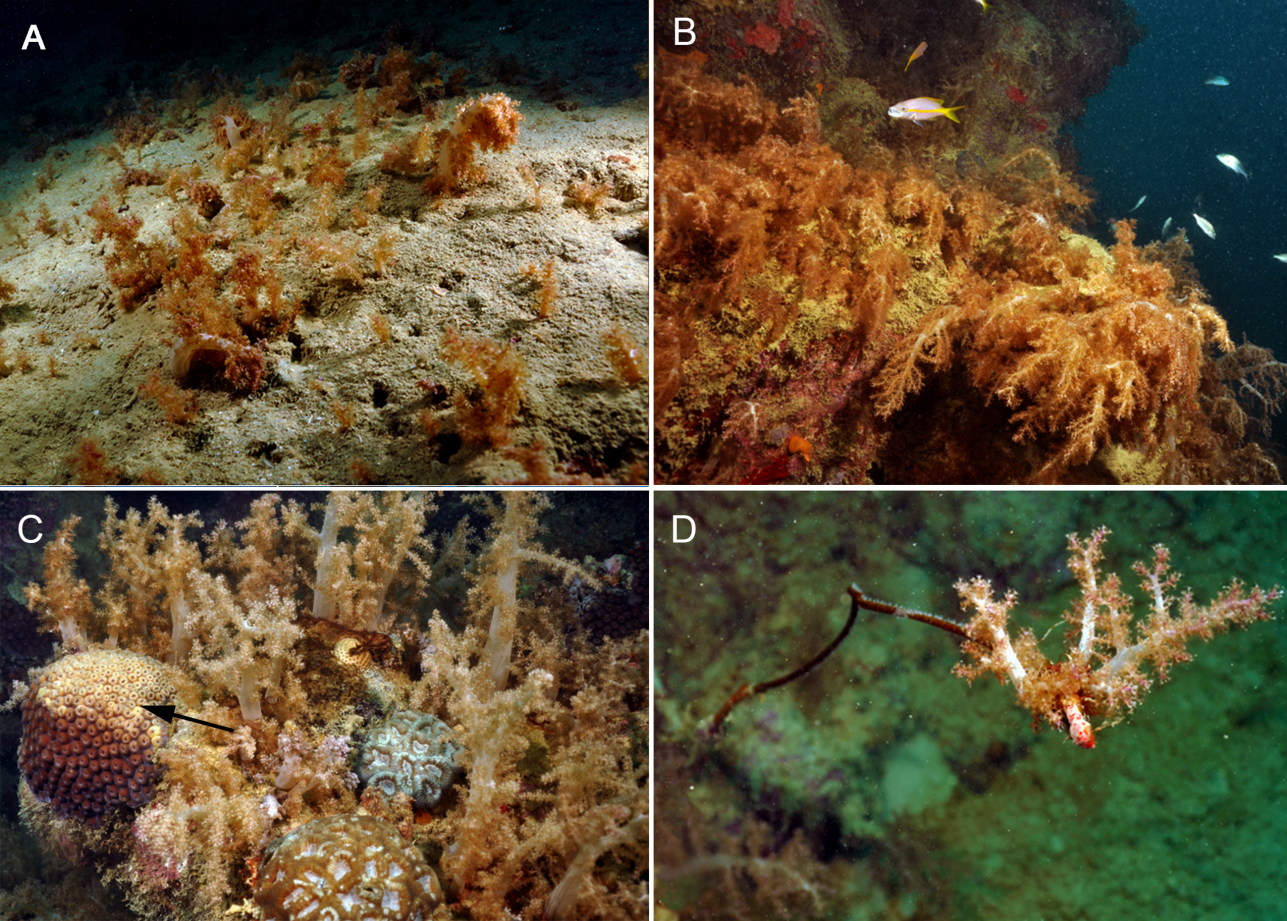
Substrate dominance and allelopathic competition of *Neospongodes atlantica* to Brazilian native corals at Timbebas reef (17°28′37.2″S, 39°01′37.2″W, 10–15 m deep). (A) Aggregation of *N. atlantica* on sandy bottom in the Abrolhos region, Bahia. (B) *N. atlantica* colonies dominating a reef edge on Abrolhos reefs. (C) Bleaching and partial necrosis caused by *N. atlantica* on the scleractinian coral *Montastraea cavernosa* (arrow). (D) *N. atlantica* covering a black-coral colony (*Stichopathes* sp.). All photos by RB Francini-Filho.

Conventional SCUBA, ROV and technical dive surveys revealed extensive aggregations of *N. atlantica* dominating deeper soft-substrata (mesophotic depth) on the continental shelf (Fig. 2A; ESM 2, ESM 3) and shallow reef-walls surrounded by sandy bottoms (Figs. 2B–2D). All observed contact interactions were harmful to other coral species (Figs. 2C–2D). Although *in situ* evidence of OFs was only obtained for the surveyed sites, they may follow the distribution range of *N. atlantica*. The Cabeço, for example, area with the largest OF found during our surveys had nearly 1 km^2^ surveyed but the OF limits were not reached.

### Morphology

**Figure 3:**
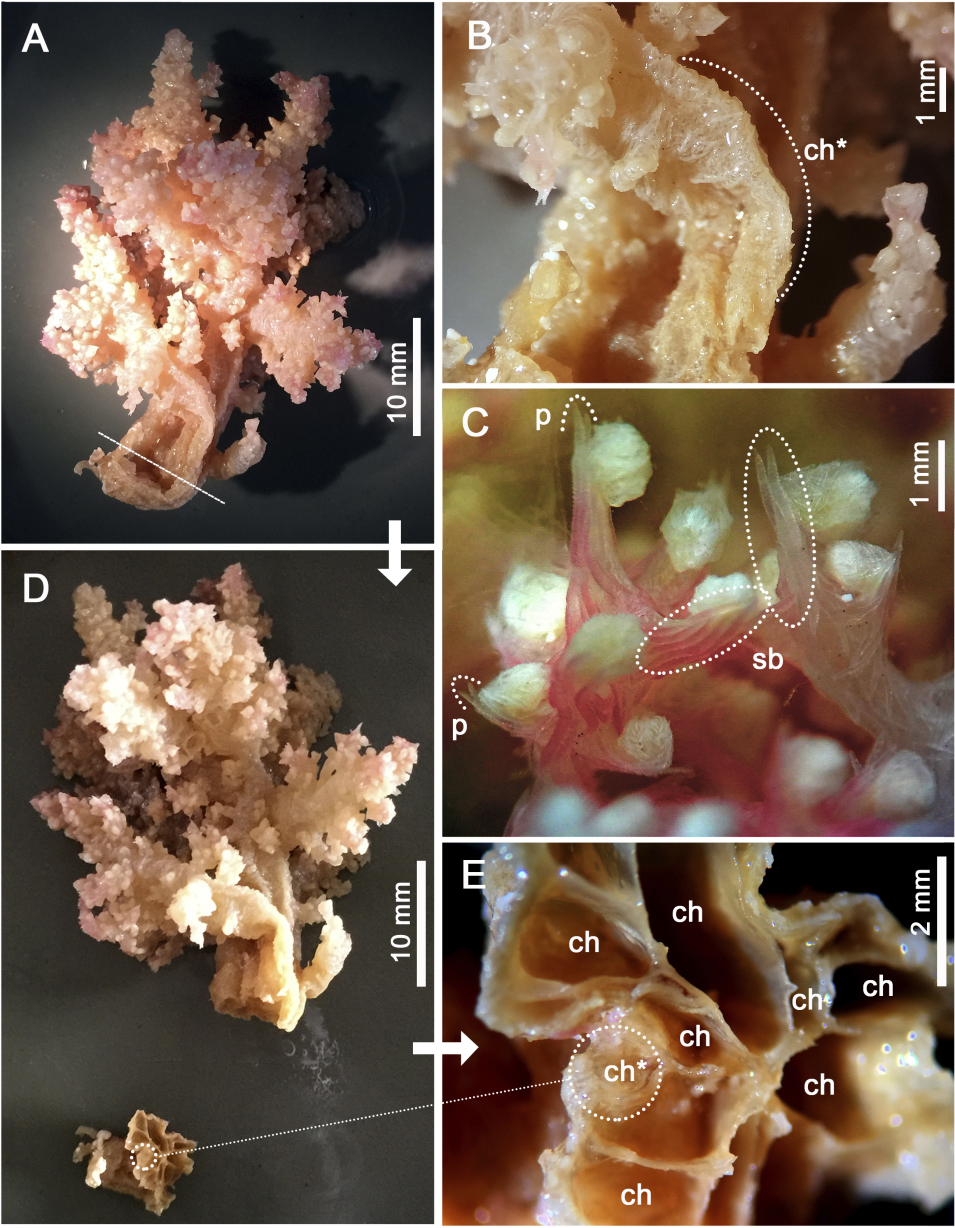
Main distinctive morphological characters in *Neospongodes atlantica* (MOUFPE 431). (A) Entire colony. (B) View of the stalk with a central channel, in which sclerites occur in a higher density. (C) View of anthocodia on a branch tip showing projecting supporting bundles. (D) Colony with its stalk transversally sectioned, showing longitudinal channels. (E) Colony channels in detail. Abbreviations: ch - channel; p - projecting tip of a spindle; sb - supporting bundle of sclerites; and ch* - pseudo-axis.

**Figure 4:**
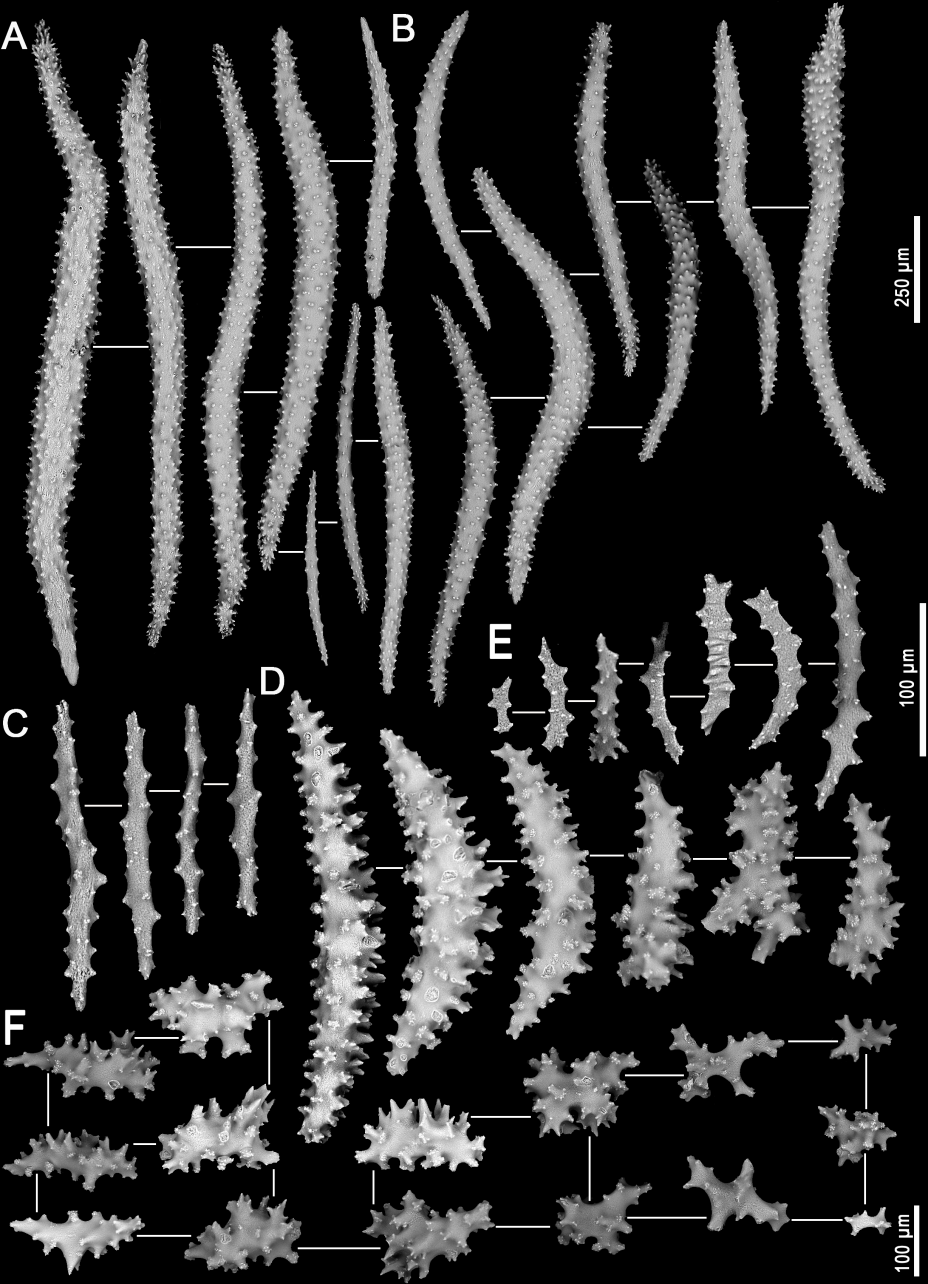
Sclerites of *Neospongodes atlantica* Kukenthal, 1903 (MOUFPE 439). (A) Spindles of the supporting bundle; (B) spindles of the anthocodial points; (C) flattened rods of the crown region; (D) spindles of the interior of the stalk; (E) flattened rods of the adaxial portion of the polyp; (F) sclerites of the surface of the stalk.

Assessment/identification of museum samples yielded 60 records of *Neospongodes atlantica* (between ∼1°S and 19°S, from 1 to 75 m depths; Table S2), most of which have not been published before. The species has highly flexible and arborescent colonies rarely taller than 100 mm, with branches standing upwards (Fig. 3A), resulting in a width of about 60 mm. Colonies coloration varies from white, pink to pale brown, and are attached to substrate through slender coenenchyme basal expansions. The polyps occur on the branches, but most are distally concentrated, all with a projecting supporting bundle of sclerites in the form of spindles of up to 1.7 mm in length (Figs. 3C, 4A, Fig. S1). The colony stalk is up to 20 mm wide, have no polyp, and is composed of several longitudinal channels separated by thin walls, in which spindles are common (Figs. 3D, 4D). In most examined samples, one of the channels shows a higher concentration of such sclerites, forming an irregular pseudo-axis (Figs. 3B, 3E). It is important to mention that colony measurements given herein correspond to ethanol-preserved samples, which are mostly contracted. Polyps with an anthocodial armature formed by spindles arranged in chevrons of up to six lines (0.5 to 1.0 mm long), become smaller and more linear towards the tentacles (0.15 to 0.30 mm long) (Figs. 4B, 4C, 4E). Spindles on the supporting bundles often curved and pink-colored (0.3 to 1.2 mm).

### Molecular and phylogenetic analysis

Molecular analysis resulted in sequences of 662 bp long for the *mtMutS*, 707 bp for the *COI* and only 322 bp for the *28S* marker. The final alignment, consisting of three concatenated genes (*mtMutS*, *COI*, and 28S), included 103 taxa and is 2,181 bp long, of which 1,275 of the 1,840 variable sites are parsimony informative. The best-fit models for the final alignment were the GTR+G (*mtMutS*), HKY+G (*COI*), and K80+I+G (*28S*). Pairwise distance between *Stereonephthya* cf. *cundabiluensis* from Palau (GenBank accession numbers: KF915783 and KF955259) and *N. atlantica* resulted in differences smaller than 0.5% within mitochondrial markers. However, when comparing the *28S* dataset, the specimen identified as *Dendronephthya* sp. from Palau (GenBank accession number: KF915355) is more similar to sequenced *N. atlantica* (*p*-distance of 0.094) than to *Stereonephthya* species, which have a pairwise distance between 0.11 to 0.29.

**Figure 5:**
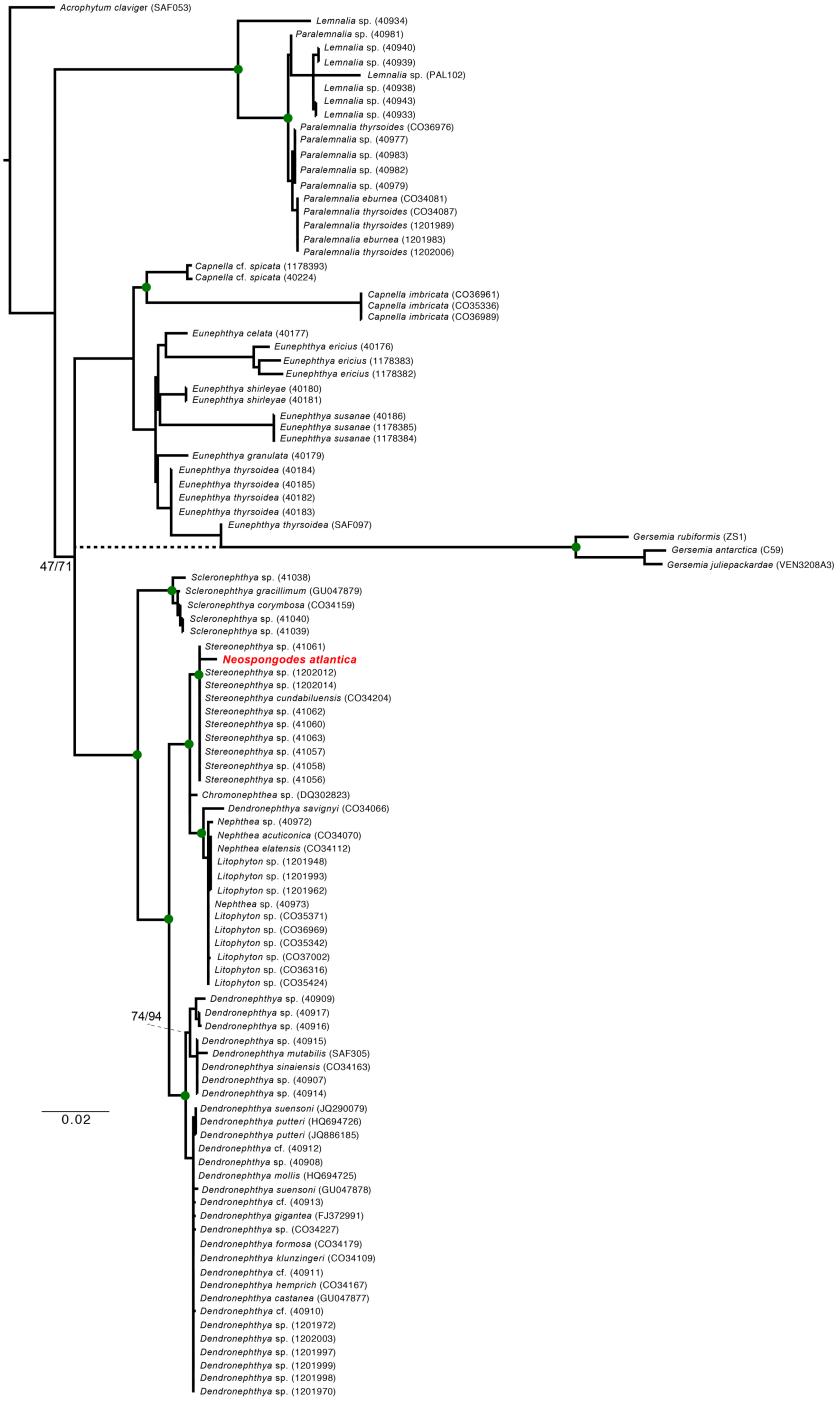
Maximum Likelihood and Bayesian inference phylogenetic reconstructions of the family Nephtheidae based on the extended Octocorallia barcoding genes (*mtMutS* + *COI + 28S*) concatenated data. The Brazilian specimen of *Neospongodes atlantica* sampled from Recife is highlighted in red. Green circles on nodes indicate supports over 80% for ML and posterior probability of 100 (BI). Dashed branch indicate the *Gersemia* clade leading branch retrieve in BI. Genbank sequences of *Nephthea* (40972, 40973, CO34070, CO34112) correspond to *Litophyton*, recently synonymized.

The ML and BI (Fig. 5) phylogenies based on the concatenated alignment unequivocally retrieved a clade containing all *Stereonephthya* spp. and *N. atlantica* (95-ML; 100-Posterior Probability [BI]). This clade is close related to that formed by *Litophyton*, *Nephthea*, and *Dendronephthya savignyi* (97-ML; 100-PP). Remaining *Dendronephthya* representatives tested (30 specimens; 98-ML and 100-PP) were recovered as a sister clade to the aforementioned groups. Most of the remaining genera included in our phylogeny appears to be monophyletic, the exception being *Paralemnalia* sp. (40981) that is more closely related to *Lemnalia* spp. than to its congeners. The only discrepancy between ML and BI recovered topologies was the position of the *Gersemia* clade that although monophyletic, was retrieved diverging from *Eunephthya thysoides* in the ML and as polytomy in the BI topology (see dashed line in Fig. 5).

### Species distribution models

A PCA showed that more than 99% of the environmental variance was explained by groups of two, three, or four variables (Data S2). Pearson correlation selecting eight variables (Data S2), suggested that 64 out of the 93 possible combinations have auto-correlation above 0,8. Thus, we built 24 datasets with two variables, 28 with three variables, and 12 with four (Data S2).

**Figure 6:**
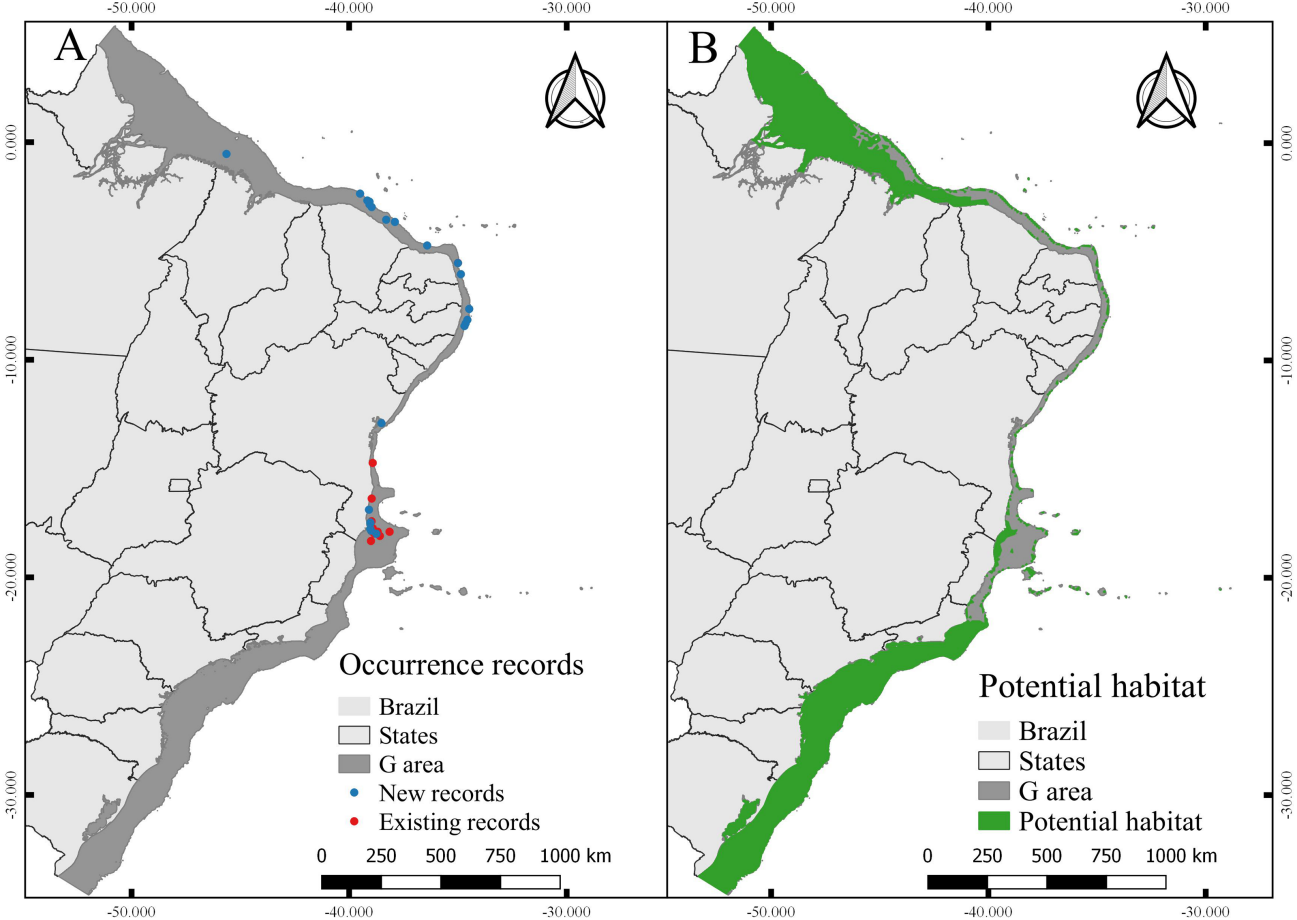
Occurrence records and current potential habitat. (A) Modeling records of *Neospongodes atlantica* (new) and evaluation records (previously existing records). (B) Potential habitat area for *N. atlantica* in the Brazilian coast, from intertidal zone to shelf-break.

After filtering procedures, a total of 23 records were used for model building (new records) and 12 records were used for model testing (independent records) (Fig. 6). The evaluation of the models calibrated in **M** resulted in 15 candidates according to omission rates and AICc (Data S3) (Table 3). The final model (from those projected in G) was built using maximum temperature (57.2% of contribution) and maximum silicate (42.8% of contribution), RM = 0.6 and FC = pt.

**Table 3:** Candidate models calibrated in **M**.

Criterion	Number of models
All candidate models	53,824
Statistically significant models	24,182
Models that meet the omission rate criteria	34,193
Models that meet the AICc criteria	34
Statistically significant models that meet the omission rate criteria	6,169
Statistically significant models that meet the AICc criteria	24
Statistically significant models that meet the omission rate and AICc criteria	15

**Figure 7:**
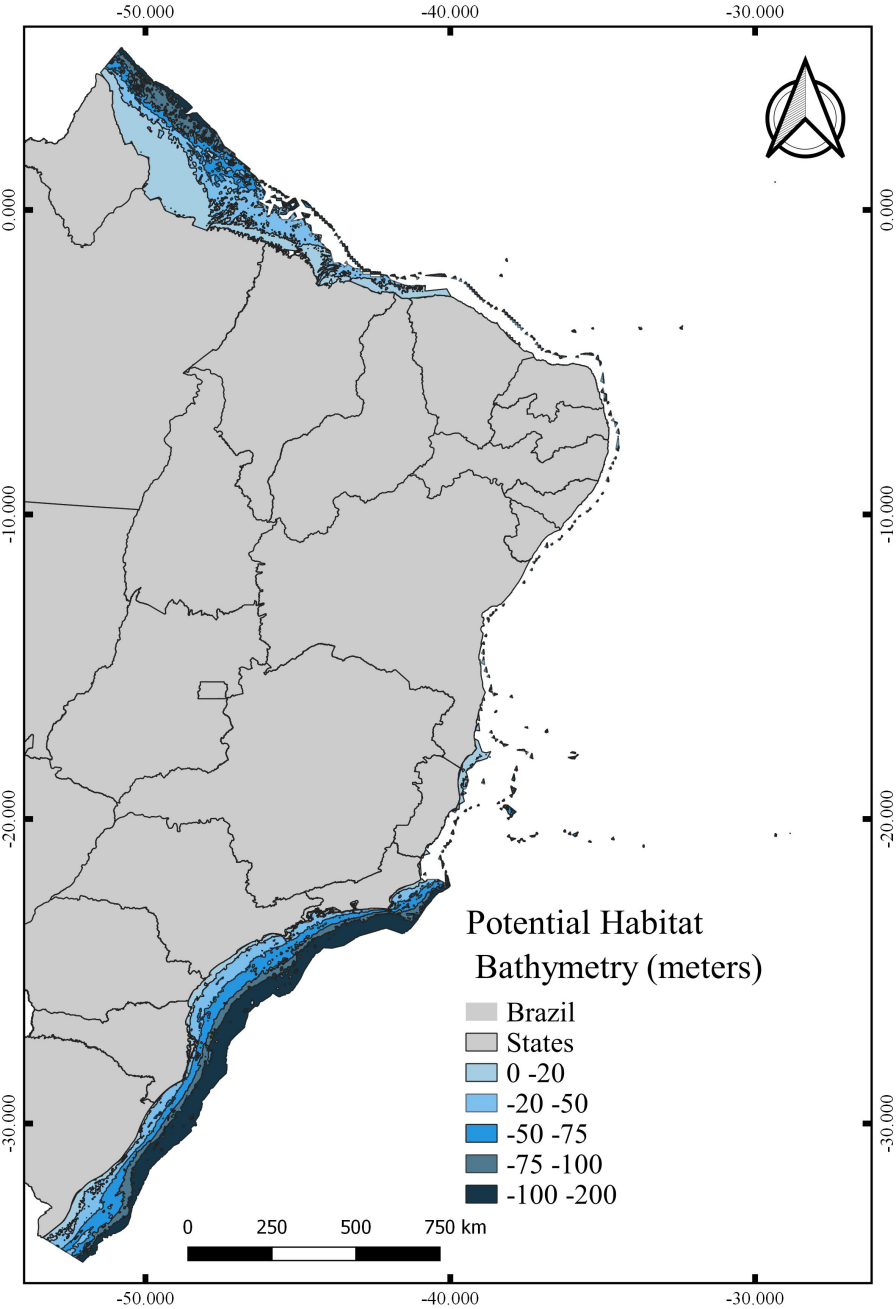
Potential habitat for *Neospongodes atlantica* at five different depths within the Brazilian coast from the intertidal zone to the end of the continental shelf.

The total area with PH for *N. atlantica* in the examined area is approximately 553,015.5 km^2^, occupying 73.07% of the G area (Fig. 6). The use of buffers around the occurrence records to calibrate the model can reduce uncertainties when such model is extrapolated to new areas beyond the calibration area (Ficetola et al., 2014; Fulgêncio-Lima et al., 2021; Machado-Stredel, Cobos & Peterson, 2021). Bathymetric analyzes showed that 29.4% of the PH is between 0 and 20 m deep (162,670 km^2^), 24.2% from 20 to 50 m (134,382 km^2^), 16.6% between 50 and 75 m (91,943.9 km^2^), 10.9% from 75 to 100 m (60,674.6 km^2^), and 18.6% around 100 and 200 m (103,345 km^2^) (Fig. 7).

**Figure 8:**
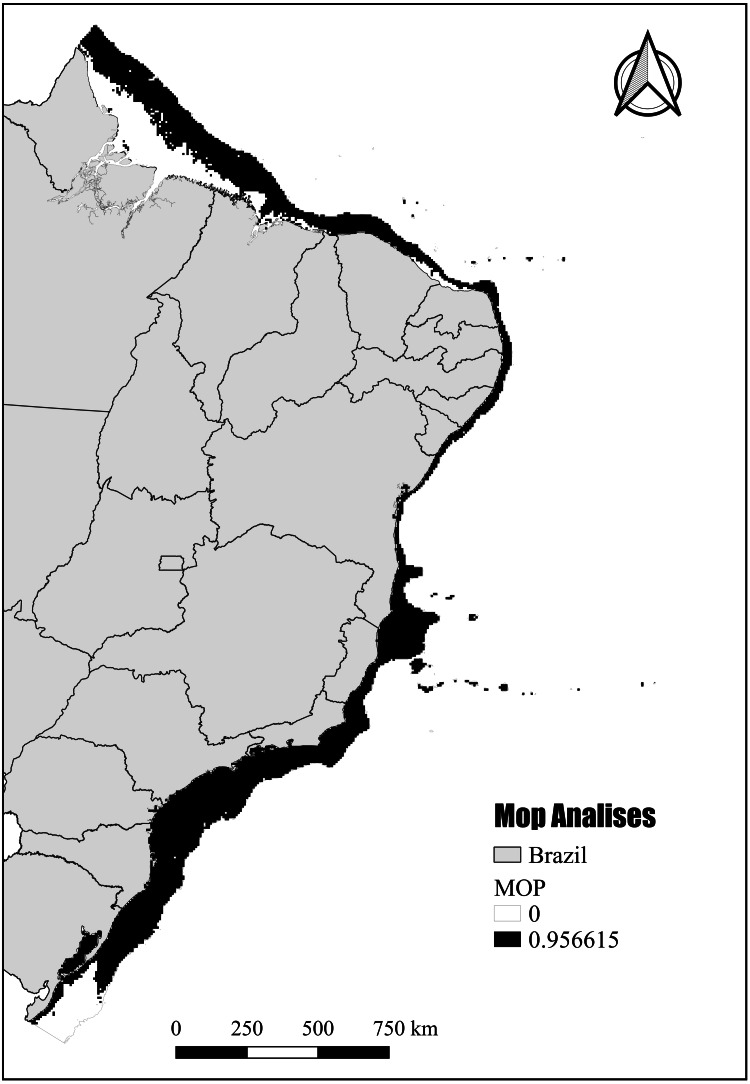
Potential habitat end mobility-oriented parity analysis (MOP). Assessment of the risk of extrapolation of M in G.

The mobility-oriented parity analysis (MOP) revealed that most of the areas with PH showed a high risk of strict extrapolation within the Brazilian continental shelf (G) (Fig. 8). These G areas have values outside the environmental range of the calibration area (M). However, the regions in the southernmost and northernmost (Amazon reefs) limits showed very low or zero risk of extrapolation (Fig. 8).

## Discussion

### *Neospongodes atlantica* and the systematics of nephtheids

Nephtheids have been classified mostly based on morphological data. Nonetheless, despite morphology being essential for the correct identification of octocorals, due to plasticity and homoplasy, it is nothing less than challenging to differentiate nephtheid genera and species (Ofwegen & Groenenberg, 2007). Therefore, improved morphological data acquisition techniques, such as sclerite analysis using SEM, combined with genetic data, suggested that, not only Nephtheidae, but many octocoral families and genera were unnatural groupings resulting from poor identifications/descriptions (Verseveldt, 1983; Ofwegen, 2005; Ofwegen & Groenenberg, 2007). For example, *Dendronephthya* Kükenthal (1905), *Litophyton* Forskål (1775), and *Stereonephthya*, genera that were recovered as a well-supported clade in the present study, are morphologically complex and, in general, poorly studied, as discussed in the most recent and comprehensive revisions (Ofwegen & Groenenberg, 2007; Ofwegen, 2020). Although this grouping has received high statistical support, their morphological complexity is reflected in the resulting phylogeny. Exceptions were the genera *Capnella*, *Lemnalia*, *Paralemnalia*, *Eunephthya*, and *Gersemia*, in which morphology classifications correspond to well-supported molecular clades (*e.g.*, McFadden et al., 2014).

In their nephtheid molecular analysis, Ofwegen & Groenenberg (2007) found two main clades: (I) *Chromonephthea*/*Stereonephthya*; and (II) *Nephthea*/*Litophyton*. As a result, *Litophyton* and *Nephthea* were later merged (Ofwegen, 2016; Ofwegen, 2020). Although our results corroborate that *Nephthya savignyi* Ehrenberg (1834), the type species of *Dendronephthya*, belongs to *Litophyton* (Ofwegen & Groenenberg, 2007), most of the available *Dendronephthya* sequences falls as a sister group of their Clades I and II (Fig. 5), indicating that a further revision of *Dendronephthya* is also needed. Despite several uncertainties regarding the diagnostic morphological characters in nephtheids (Verseveldt, 1983; Ofwegen & Groenenberg, 2007), most of its genera appears to be well-resolved. The recalcitrant taxa are limited to some species in need of additional assessments, such as *Neospongodes atlantica*, which was recovered herein within a clade composed solely by *Stereonephthya* representatives (Fig. 5).

Overall, the distinction between *Stereonephthya* and *Neospongodes* is based on weak evidence: the presence of an accumulation of sclerites in the center of the stalk of the latter, called herein pseudoaxis (see Verseveldt, 1983; Ofwegen & Groenenberg, 2007). The nature of such an axis was already discussed in the literature and it is known to be convergent in *Neospongodes*, *Scleronephthya*, and in several *Dendronephthya* and *Stereonephthya* representatives (Verseveldt, 1983). In addition, colony shape is not a good generic diagnostic character (Ofwegen, 2016), as shown by *Litophyton savignyi* (=*Nephthya savignyi*). Thus, so far, the polyp armature along with its highly projecting supporting bundle, despite convergent in some genera, seems to be consistent in *Stereonephthya* and *Neospongodes* (Fig. 3; Fig. S1). The only uncertainty of such character remains on *S. portoricensis*, as Bayer (1961) illustrates it with projecting supporting bundles (Fig. S1), a feature reiterated by Verseveldt (1983) although no signs of it is seen in his detailed drawing of the antocodium (Fig. S1). Both *Stereonephthya* and *Neospongodes* share well-developed supporting bundles of the polyps with strong projecting tip; ventral side of the polyp stalk has small tiny rods; and strong dorsal points but ventral points poorly supported or lacking (Utinomi, 1954). Thus, based on the current molecular and morphological evidence, there are no reasons to keep both genera separated, as previously suggested by Ofwegen & Groenenberg (2007).

### Octocoral forests and their extensive distribution

Our results fill a considerable gap in the distribution of *N. atlantica* along the Brazilian coast, especially in between the records from Ceará (3°26′S and 38°08′W) and Bahia states (∼12°S to ∼17°S, see Castro, Medeiros & Loiola (2010)). In this area, *N. atlantica* appears as a minor element in the shallow reef environments, especially in the Abrolhos regions (Laborel, 1969; Castro et al., 2005; Leão & Kikuchi, 2000). However, it forms dense aggregations on soft bottoms at mesophotic depths (Moura et al., 2013). These results contrast with the Indo-Pacific region, where the majority of shallow water nephtheids are associated with zooxanthellae (*Litophyton*, *Lemnalia*, *Capnella*, *etc*.) (Fabricius & Alderslade, 2001; Schubert, Brown & Rossi, 2017), and only a few such depth generalists are found below 25–30 m. Nonetheless, the symbiotic taxa of this family are also found in mesophotic depths, on both hard and soft substrates (*e.g.*, *Umbellulifera*). Eastern nephteids may form either monospecific carpets or diverse aggregations with as many as 31 species per 600 m^2^ (Bayer, 1961; Tursch & Tursch, 1982) and, in reef communities, they may total a biomass 10 times higher than that of scleractinian corals (Benayahu & Loya, 1987; Tursch & Tursch, 1982).

Despite being considered a typical reef species, the mesophotic OFs found in Brazil suggest that *N. atlantica* should be referred to as a soft-bottom species, as its occurrence in reefs is restricted to reef-sand interfaces and reef-walls influenced by the amount of suitable habitat (soft bottom) around them, as recorded for the Cabeço and Timbebas Reef (Fig. 2). Such a behavior is shared by other nephtheid genera such as *Dendronephthya* and *Umbellulifera*, both considered typical sand dwellers (Fabricius & Alderslade, 2001).

Although inhabiting the photic zone, *N. atlantica* is an azooxanthellate species that, similarly to the Indo-Pacific *Dendronephthya* and *Stereonephthya* (see Fabricius & McCorry, 2006; Ofwegen & Groenenberg, 2007), appears to have a preference for low-light exposition. Whereas light availability limits the occurrence of the majority of Indo-Pacific zooxanthellate soft-corals to shallow and well-lit depths Tursch & Tursch, 1982), *N. atlantica* forms aggregations to up to 95 m deep (Pérez, Neves & Oliveira, 2011). In terms of substrate, based on data available from museum samples and the Abrolhos stations from Moura et al. (2013), *N. atlantica* appears to have a preference for sandy bottoms with high CaCO3 and moderate silt/mud concentrations. Given its success in such substrata, reef communities close to sediment discharges (*e.g.*, dredging or river discharges) should be carefully monitored. In that context, most areas modeled as PSH for *N. atlantica* were found at the northernmost and southernmost limits of the Brazilian Continental Shelf, where no record of the species are yet known but great quantities of sand, silt, and mud dischargers occur from the Amazon and La Plata rivers respectively. Although these unknown occurrence areas might be considered as an accurate representation of the spatial extent that provides habitable conditions for the targeted species (Peterson, Papeş & Soberón, 2008), the MOP analysis indicated that most of the PH in those areas point to the risk of high extrapolation. Such areas have values outside the climatic range of the calibration area (see Raghavan et al., 2019) and, therefore, caution is required when assessing the probability of *N. atlantica* occurrence in these areas.

Taking into consideration that (i) morphological differentiation between *Neospongodes* and *Stereonephthya* (if it really exists) is yet to be proposed, (ii) *N. atlantica* is molecularly identical to Indo-Pacific *Stereonephthya*, and (iii) *N. atlantica* is the only representative of the family in the Southwestern Atlantic (*Chromonephthea braziliensis* has been reported as non-indigenous (NIS) in Brazil (see Ofwegen (2005))), we consider it inappropriate to keep both genera separated. Therefore, according to the International Code of Zoological Nomenclature, all species names currently associated with *Stereonephthya* should be moved to *Neospongodes,* as the latter has date priority in relation to the former. Unfortunately, the data presented herein suggest that *N. atlantica* was probably introduced in the Southwestern Atlantic as earlier as 1903 (*i.e.*, the year of its description) either by Portuguese or Dutch vessels transiting within the marine trade routes (Ebert, 2003; see also Morandini et al., 2017). Otherwise, the alternative hypothesis would be a common origin shared with *Stereonephthya portoricensis*, a species found in mesophotic to deep Caribbean waters (Cairns, 2017) in a previous Atlantic-Pacific-split scenario. To test these hypotheses, it would be necessary to sequence *S. portoricensis*, which is unavailable at this moment.

Considering the hypothesis that *N. atlantica* is a NIS is alarming, even though it would not be the first long-hidden introduced species into Brazilian waters. For example, the once thought native octocoral *Carijoa riisei* and the bivalve *Perna perna* have been suggested to be introduced a long time ago (Grigg, 2003; Concepción et al., 2010; Pierri, Fossari & Magalhães, 2016; Galván-Villa & Ríos-Jara, 2018; Lima et al., 2018), even though there is not a consensus in the case of the bivalve (Calazans et al., 2021). Some of the problems of NIS are related to the fact that they can become invasive, altering the ecosystem function and, therefore, causing biodiversity loss and economic impacts (Bax et al., 2003; Chakraborty, 2019; Carpinelli et al., 2020). In the case of *N. atlantica*, its populations have apparently increased recently on soft (as previous expeditions did not report it) and hard-substrate. Nonetheless, as most of the records presented herein are from mesophotic depths (see Table S2), its spread along the Brazilian continental shelf remained mostly unnoticed. In our field observations, it was found to cause tissue necrosis in one of the main reef-building scleractinian species in Brazil, the great-star-coral *Montastraea cavernosa* (Fig. 2), a species that is abundant at mesophotic depths (Francini-Filho et al., 2019). Many soft corals, including nephtheids, bear diverse secondary metabolites, which makes them unpalatable, as well as strong spatial competitors (Tursch, 1982; Sammarco, La Barre & Coll, 1987; Allam et al., 2021). Terpenoids either in tissue or in the surrounding water make these soft-corals superior competitors even without direct contact with other organisms (allelopathy), such as scleractinian corals (Coll et al., 1982; Fabricius, 1997). For example, an invasive xeniid octocoral has invaded the Venezuelan Caribbean and, more recently, its abundance has surpassed that of all other benthic taxa in that region (Ruiz-Allais, Benayahu & Lasso-Alcalá, 2021). Invasive xeniids and other non-indigenous octocoral species from other families have also been reported in Brazil (Mantelatto et al., 2018; Carpinelli et al., 2020; Menezes et al., 2021), raising concerns on the fate of invaded localities biodiversity and, consequently, ecosystem function. Therefore, we recommend that populations of *N. atlantica* should be monitored in order to keep track of its spread and eventual invasiveness in Brazilian marine environments, along with other recently aquarium-introduced octocorals (Mantelatto et al., 2018; Carpinelli et al., 2020).

## Additional Information and Declarations

### Field Study Permissions

The following information was supplied relating to field study approvals (i.e., approving body and any reference numbers): ICMBio approved the study (SISBIO 15691-1, SISBIO-11709-1).

### DNA Deposition

The following information was supplied regarding the deposition of DNA sequences: The sequences are available in the Supplementary Files and at Genbank: OL684635, OL684634, OL684632 and OL684633.

### Data Availability

The following information was supplied regarding data availability: The raw data are available in the Supplementary Files. The *in situ* video records of soft coral beds are available at figshare: Cordeiro, Ralf; Carpinelli, Ágatha; Francini-filho, Ronaldo; Neves, Bárbara; Pérez, Carlos; Oliveira, Umberto; et al. (2022): Supplemental Video 1. figshare. Media. https://doi.org/10.6084/m9.figshare.21534636.v2. Cordeiro, Ralf; Carpinelli, Ágatha; Francini-filho, Ronaldo; de Moura Neves, Bárbara; Pérez, Carlos Daniel; Oliveira, Umberto; et al. (2022): Supplemental Video 2. figshare. Media. https://doi.org/10.6084/m9.figshare.21539517.v2. Cordeiro, Ralf; Carpinelli, Ágatha; Francini-filho, Ronaldo; de Moura Neves, Bárbara; Pérez, Carlos Daniel; de Oliveira, Umberto Diego Rodrigues; et al. (2022): Supplemental Video 3. figshare. Media. https://doi.org/10.6084/m9.figshare.21539613.v1.

## Supplemental Information

Supplemental Information 1 msh1 alignment (Genbank: OL684635)

Supplemental Information 2 COI alignment (Genbank: OL684634)

Supplemental Information 3 28S alignment (Genbank: OL684632 and OL684633)

Supplemental Information 4 Sequences used for *Neospongodes atlantica* phylogenetic reconstruction and their respective GenBank Accession numbers

Supplemental Information 5 Records used to build models Previous and new geographic records of *Neospongodes atlantica*.

Supplemental Information 6 Raw data and analyses PCA1, PCA2, possible combinations of the variables, Pearson correlation, set of variables used

Supplemental Information 7 Models Results of calibrations of the models, evaluation of the final models

Supplemental Information 8 Museum records of *Neospongodes atlantica* MOUFPE-CNI: Museu Oceanográfico Petrônio Alves Coelho (Cnidaria collection), Recife, PE, Brazil; MNRJ: Museu Nacional, Rio de Janeiro, RJ, Brazil. Depth (m), Expedition or R/V are provided.

Supplemental Information 9 Illustrations of anthocodial armatures in Stereonephthya spp Species of *Stereonephthya* Kükenthal, 1905 for which good llustrations of the anthocodia are available. Sources: Verseveldt (1966, 1973), Kükenthal (1904), Thomson & Henderson (1906). H and I correspond to illustrations of anthocodium and fragment of *S. portorricensis* (Hargitt, 1901) by Verseveldt (1983) and Bayer (1961), respectively. Images out of scale.
