# Supplementary material for: Neospongodes atlantica, a potential case of an early biological introduction in the Southwestern Atlantic
Source: PeerJ. 2022 Dec 15;10:e14347. doi: 10.7717/peerj.14347 (PMC9760029; doi:10.7717/peerj.14347)
Supplement: Supplemental Information 9 — Species of Stereonephthya Kükenthal, 1905 for which good llustrations of the anthocodia are available. Sources: Verseveldt (1966, 1973), Kükenthal (1904), Thomson & Henderson (1906). H and I correspond to illustrations of anthocodium and fragment of S. portorricensis (Hargitt, 1901) by Verseveldt (1983) and Bayer (1961), respectively. Images out of scale. [file peerj-10-14347-s009.pdf]

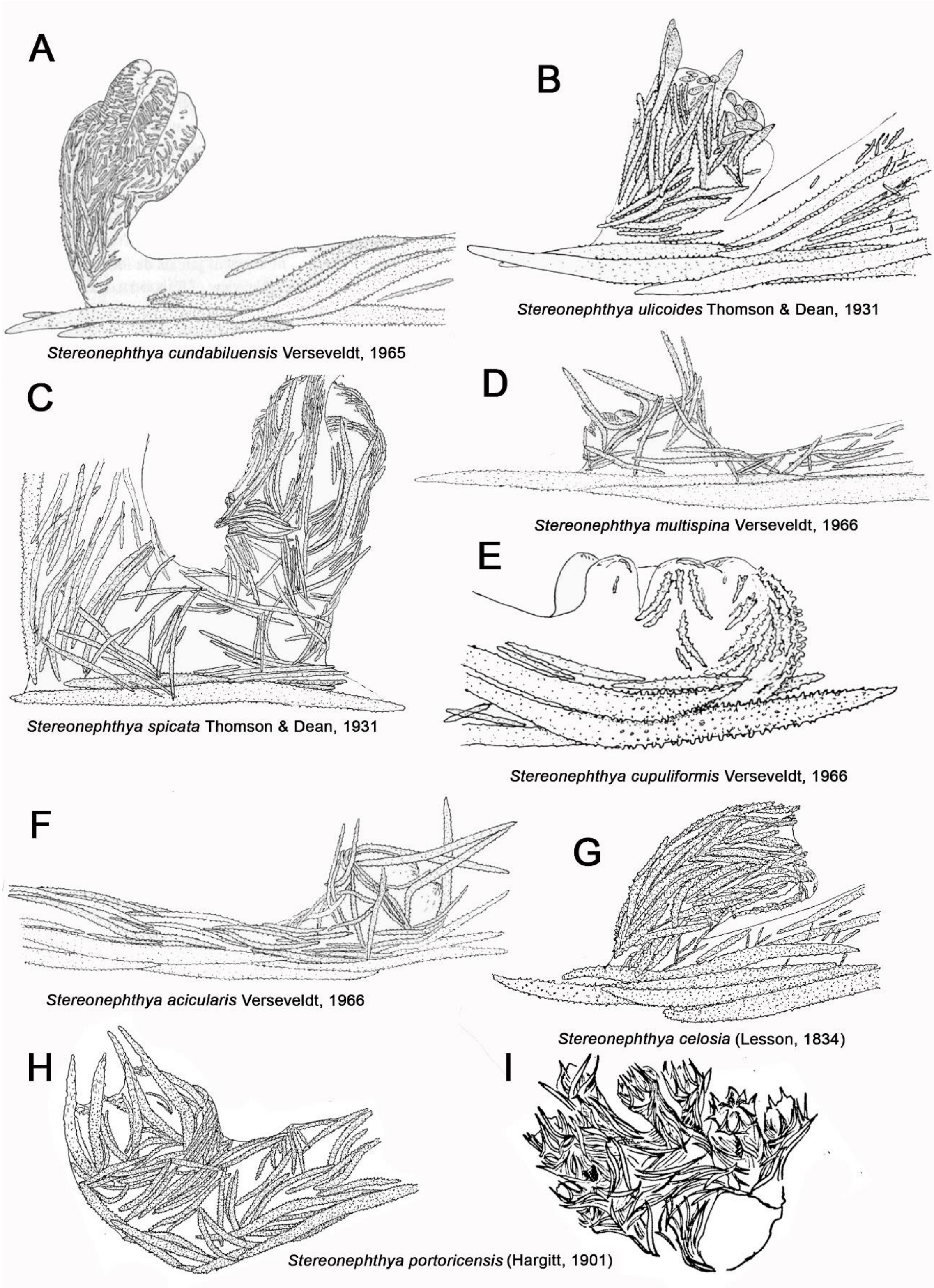

Figure S1. Species of *Stereonephthya* Kükenthal, 1905 for which detailed illustrations of the anthocodia are available. Sources: Verseveldt (1966, 1973), Kükenthal (1904), Thomson & Henderson (1906). H and I correspond to illustrations of the anthocodium and fragment of *S. portorricensis* (Hargitt, 1901) by Verseveldt (1983) and Bayer (1961), respectively. Images out of scale.

Thomson & Dean, 1931

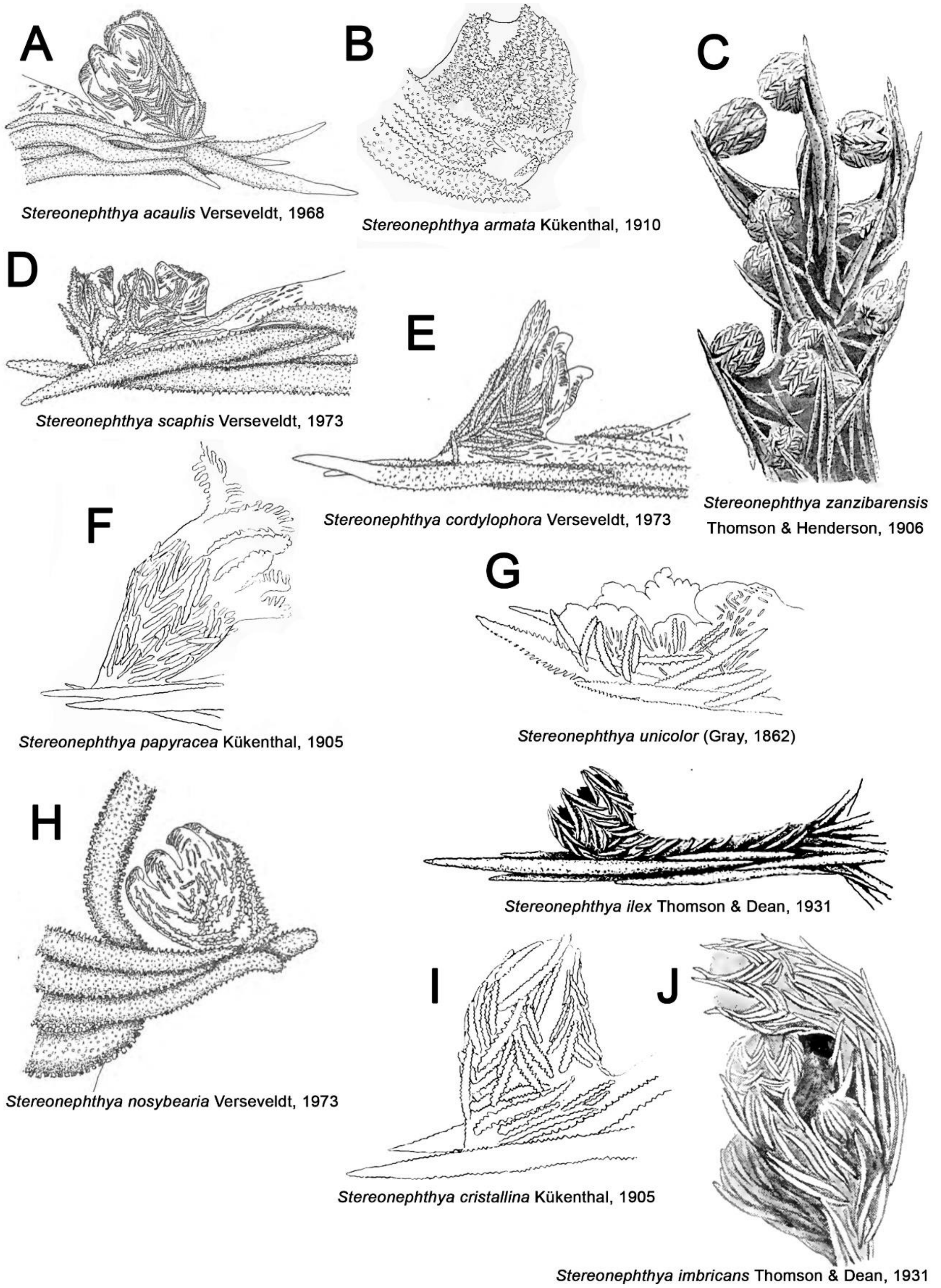

Figure S2. Species of *Stereonephthya* Kükenthal, 1905 for which detailed illustrations of the anthocodia are available. Sources: Verseveldt (1966, 1973, 1983), Kükenthal (1904), Thomson & Henderson (1906). Images out of scale.

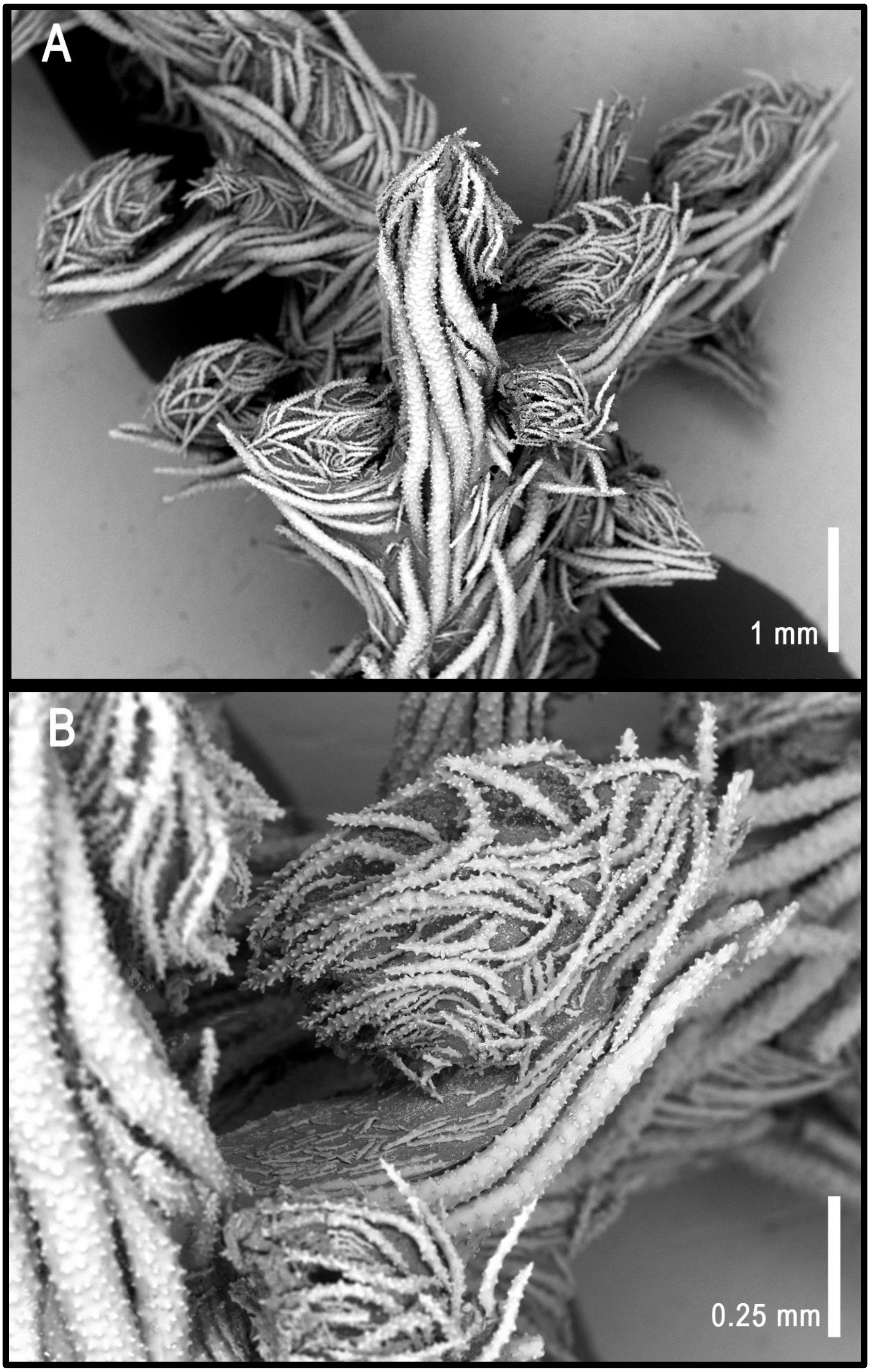

Figure S3. Detailed view of the anthocodia in *Neospongodes atlantica* Kükenthal, 1903 (MOUFPE 439). A: front view of a supporting bundle of sclerites; B: side-view of a polyp, showing sclerite organization.

REFERENCES

Bayer, F. M. (1961) The shallow-water Octocorallia of the West Indian region. A manual for marine biologists. Studies on the Fauna Curacao and other Caribbean Islands, 12:1-373.

Hargitt, C.W. & Rogers, C.G. (1901) The Alcyonaria of Porto Rico. Bulletin of the U.S. Fish Commission, 20(2): 265-287.

Kukenthal, W. (1904) Uber einige Korallentiere des Roten Meeres. In: Festschrift zum siebzigsten Geburtstage von Ernst Haeckel herausgegeben von seinen Schulern und Freunden. Jenaische Denkschriften, 11:31-58.

Kukenthal, W. (1905) Versuch einer Revision der Alcyonaceen. 2. Die Familie der Nephthyiden. 2 Teil. Die Gattungen *Dendronephthya* n.g. und *Stereonephthya* n.g. Zoologische Jahrbücher. (Systematik), 21(5/6): 503-726.

Thomson, J. Arthur And W.D. Henderson.--1906. Alcyonaria. In: The marine fauna of Zanzibar British East Africa, from collections made by Cyril Crossland, M.A., B.Sc., F.Z.S., in the years 1901 and 1902. Proceedings of the Zoological Society of London, 1906(1): 393-443.

Verseveldt, J. (1966) Octocorallia from the Malay Archipelago (Part II). Biological Results of the Snellius Expedition XXII. Zoologische Verhandelingen Leiden, 80:1-107.

Verseveldt, J. (1973) Octocorallia from north-western Madagascar (Parts IIC, IIID). Proceedings, Koninklijke Nederlandse Akademie van Wetenschappen, Series C, 76(2): 140-171.

Verseveldt, J. (1983) The octocorallian genera *Spongodes* Lesson, *Neospongodes* Kukenthal and *Stereonephthya* Kukenthal. Beaufortia 33(1): 1-13.
